# Epidemiology and Outcome of Severe Sepsis and Septic Shock in Intensive Care Units in Mainland China

**DOI:** 10.1371/journal.pone.0107181

**Published:** 2014-09-16

**Authors:** Jianfang Zhou, Chuanyun Qian, Mingyan Zhao, Xiangyou Yu, Yan Kang, Xiaochun Ma, Yuhang Ai, Yuan Xu, Dexin Liu, Youzhong An, Dawei Wu, Renhua Sun, Shusheng Li, Zhenjie Hu, Xiangyuan Cao, Fachun Zhou, Li Jiang, Jiandong Lin, Enqiang Mao, Tiehe Qin, Zhenyang He, Lihua Zhou, Bin Du, for the China Critical Care Clinical Trials Group (CCCCTG)

**Affiliations:** 1 Medical ICU, Peking Union Medical College Hospital, Peking Union Medical College & Chinese Academy of Medical Sciences, Beijing, China; 2 Department of Emergency Medicine and Medical ICU, The First Affiliated Hospital of Kunming Medical University, Kunming, China; 3 Department of Critical Care Medicine, The First Affiliated Hospital of Harbin Medical University, Harbin, China; 4 Department of Critical Care Medicine, First Affiliated Hospital, Xinjiang Medical University, Urumqi, China; 5 Department of Critical Care Medicine, West China Hospital, Sichuan University, Chengdu, China; 6 Department of Critical Care Medicine, The First Affiliated Hospital of China Medical University, Shenyang, China; 7 Department of Critical Care Medicine, Xiangya Hospital, Central South University, Changsha, China; 8 Department of Critical Care Medicine, Beijing Tongren Hospital, Capital Medical University, Beijing, China; 9 Department of Critical Care Medicine, The Second Hospital of Jilin University, Changchun, China; 10 Department of Critical Care Medicine, Peking University People's Hospital, Beijing, China; 11 Department of Critical Care Medicine, Qilu Hospital of Shandong University, Jinan, China; 12 Department of Critical Care Medicine, Zhejiang Provincial People's Hospital, Hangzhou, China; 13 Department of Critical Care Medicine, Tongji Hospital of Tongji Medical College, Huazhong University of Science & Technology, Wuhan, China; 14 Department of Critical Care Medicine, Hebei Medical University Fourth Hospital, Shijiazhuang, China; 15 Department of Critical Care Medicine, General Hospital of Ningxia Medical University, Yinchuan, China; 16 Department of Emergency and Intensive Care Medicine, The First Affiliated Hospital of Chongqing Medical University, Chongqing, China; 17 Department of Critical Care Medicine, Fuxing Hospital, Capital Medical University, Beijing, China; 18 Department of Critical Care Medicine, The First Affiliated Hospital of Fujian Medical University, Fuzhou, China; 19 Emergency ICU, Ruijin Hospital, Shanghai Jiao Tong University, Shanghai, China; 20 Department of Critical Care Medicine, Guangdong General Hospital, Guangzhou, China; 21 Department of Critical Care Medicine, Hainan Provincial People's Hospital, Haikou, China; 22 Department of Critical Care Medicine, The Affiliated Hospital of Inner Mongolia Medical University, Huhhot, China; D'or Institute of Research and Education, Brazil

## Abstract

**Introduction:**

Information about sepsis in mainland China remains scarce and incomplete. The purpose of this study was to describe the epidemiology and outcome of severe sepsis and septic shock in mixed ICU in mainland China, as well as the independent predictors of mortality.

**Methods:**

We performed a 2-month prospective, observational cohort study in 22 closed multi-disciplinary intensive care units (ICUs). All admissions into those ICUs during the study period were screened and patients with severe sepsis or septic shock were included.

**Results:**

A total of 484 patients, 37.3 per 100 ICU admissions were diagnosed with severe sepsis (n = 365) or septic shock (n = 119) according to clinical criteria and included into this study. The most frequent sites of infection were the lung and abdomen. The overall ICU and hospital mortality rates were 28.7% (n = 139) and 33.5% (n = 162), respectively. In multivariate analyses, APACHE II score (odds ratio[OR], 1.068; 95% confidential interval[CI], 1.027–1.109), presence of ARDS (OR, 2.676; 95%CI, 1.691–4.235), bloodstream infection (OR, 2.520; 95%CI, 1.142–5.564) and comorbidity of cancer (OR, 2.246; 95%CI, 1.141–4.420) were significantly associated with mortality.

**Conclusions:**

Our results indicated that severe sepsis and septic shock were common complications in ICU patients and with high mortality in China, and can be of help to know more about severe sepsis and septic shock in China and to improve characterization and risk stratification in these patients.

## Introduction

Severe sepsis and septic shock are among the main factors contributing to mortality in intensive care units (ICUs), and exhibit a significant disease burden and negative economic impact [1]–[3].

The incidence of sepsis varies among different racial and ethnic groups [4]–[7]. Between 6 and 54% of patients admitted to ICUs have severe sepsis [2], [3], [6], [8]–[10], and the mortality rate for these patients varies from 20 to 60% [6], [10]–[12], which will increase stepwise with increasing disease severity [13]. Although the mortality rate may have decreased in recent years [5], [7], the incidence of severe sepsis and septic shock is increasing, so that overall deaths are increasing [2], [4], [7]. Even death has been avoided, the patient who survives sepsis would have a significantly compromised long-term health-related quality of life than general population [1], [14].

There have been a number of studies describing the epidemiology, risk factor and outcome of severe sepsis and septic shock in different countries [2], [4], [7], [10], [11]. Yet, information about sepsis in mainland China remains scarce and incomplete. Cheng et al [3] have described the epidemiology of severe sepsis in surgical ICUs, but data concerning the epidemiology of severe sepsis/septic shock in mixed ICUs are limited. So the China Critical Care Clinical Trials Group (CCCCTG) conducted an inception cohort study to investigate the epidemiology and outcome of severe sepsis and septic shock in mixed ICUs in China.

## Patients and Methods

### Study development

This was a secondary analysis of a prospective cohort study aiming to describe the demographics, case mix, interventions, and clinical outcome of critically ill patients admitted to ICUs in Mainland China and performed from 1 July 2009 to 31 August 2009 in 22 ICUs [15], so the data in the current study were collected prospectively but the analysis was done retrospectively. The participating ICUs were members of the CCCCTG and located in different provinces of China. The detailed characteristics of those ICUs, such as number of ICU beds, types of ICU, number of intensivists and nurses, and number of admissions in 2009 are showed in table 1. This study was approved by the institutional review board of Fuxing hospital (Number: 2009FXHEC-KY032), and the need for informed consent was waived. The ethical approval of Fuxing hospital was endorsed by the institutional review boards of all other participating centers (see the Appendix S1 for the full names and affiliation of participating hospitals) before data collection.

**Table 1:** Characteristics of participating ICUs.

*	
	Participating ICUs (n = 22)
Type of hospital	
University affiliated	19
Public	3
Number of hospital beds (median, IQR)	1730 (1402–2100)
Type of ICU	
Medical/Surgical	18
Surgical	3
Medical	1
Number of ICU beds (median, IQR)	20.5 (12.0–28.0)
Total number of intensivists	12.0 (8.5–13.8)
Total number of nurses	33.5 (26.3–45.0)
Total ICU admissions in 2009 (median, IQR)	791 (446–1353)
Hospital mortality (%)	12.1 (8.2–18.7)

ICU, intensive care unit; IQR, interquartile range.

We used a case report form (CRF) to collect data. Every participating ICU nominated a study coordinator who was responsible for screening and enrollment of patients and data collection. The CCCCTG data monitoring team was responsible for auditing the integrity of data.

### Selection of participants, data collection, and definitions

During the study period, all admissions of participating ICUs were screened for eligibility. Patients less than 15 years old or with an ICU length of stay (LOS) less than 24 hours were excluded. Patients with severe sepsis/septic shock at ICU admission or during ICU hospitalization were included in the study cohort, and only the first episode of severe sepsis or septic shock was counted. Patients readmitted into ICU during the same hospitalization were not screened again. The following information was recorded: demographic characteristics, admission category, comorbidities and preexisting organ insufficiency. The Acute Physiology and Chronic Health Evaluation (APACHE) II score [16] and Sequential Organ Failure Assessment (SOFA) score [17] on the first day of ICU were recorded to evaluate the severity of illness. Severe sepsis and septic shock were defined according to the American College of Chest Physicians/Society of Critical Care Medicine consensus conference definitions [18]. Acute respiratory distress syndrome (ARDS) were defined according to the American-European Consensus Conference criteria [19]. Acute kidney injury (AKI) was defined based on the Risk of renal dysfunction, Injury to the kidney, Failure of kidney function, Loss of kidney function and End-stage kidney disease(RIFLE) criteria [20]. Chronic organ failure and immunocompromise were diagnosed according to the criteria in APACHE II score. ICU-acquired infection was defined as the infection identified at least 48 hours after ICU admission, and ICU-acquired severe sepsis was defined as one occurring at least 48 hours following ICU admission. The reasons for ICU admission were based on disease categories of APACHE II scores.

### Outcome measures

All enrolled patients were followed up till death in the hospital or hospital discharge or until November 30, 2009, whichever occurred earlier. The primary outcome measure was incidence and crude hospital mortality of severe sepsis and septic shock, as well as the risk factors for death. ICU mortality, ICU LOS and hospital LOS were also assessed. Patients who were still in hospital on November 30, 2009 were deemed survivors.

### Statistical analysis

Data were analyzed using the SPSS 16.0 software program. Data are presented as mean and standard deviation (SD) for variables that exhibited normal distributions. On rejection of the normality hypothesis, we used median and interquartile range (IQR). Student's t-test for independent groups was applied to data with a normal distribution. When normality was rejected, the Mann–Whitney U-test was used for independent groups. For categorical variables the chi-square or Fisher's exact test was applied as appropriate. For determination of independent predictors for hospital mortality in severe sepsis patients, odds ratios (OR) and respective 95% confidence intervals (CIs) were estimated by means of multivariate logistic regression analysis. Variables including demographics, underlying diseases, severity of illness, admission status, and complications were entered into the model if p<0.2 in univariate analysis. The Hosmer-Lemeshow test was used to assess the calibration of the regression model. All comparisons were unpaired and all tests of significance were two-tailed. A p value <0.05 was considered statistically significant.

## Results

### Population characteristics and incidence of severe sepsis and septic shock

**Figure 1:**
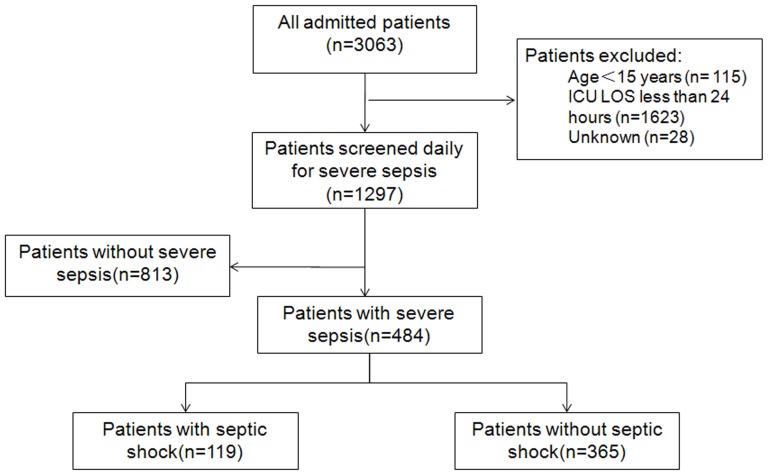
Flow diagram of enrolled patients and their outcome. ICU, intensive care unit; LOS, length of stay.

We screened 3063 admissions during the study period and 1297 patients (42.3%) were enrolled (Figure 1). A total of 484 patients developed severe sepsis or septic shock, including 336 males (69.4%), and their median age was 66 (interquartile range [IQR], 51–77) years. More than half of the patients were admitted into ICU because of respiratory diseases (53.5%), and two-thirds (67.4%) had at least one underlying disease or chronic organ system dysfunction. The median APACHE II score was 21 (IQR, 16–27), and the median SOFA score on ICU day 1 was 7.5 (IQR, 5–10). Further baseline characteristics are shown in Table 2.The incidence of severe sepsis and septic shock was 37.3 per 100 ICU admissions.

**Table 2:** Characteristics and outcome of patients with severe sepsis.

Variables	All patients (n = 484)	Survivors (n = 322)	Non-survivors (n = 162)	*P* Value
Age, median (IQR)	66 (51–77)	62 (47–74)	71 (56–80)	<0.001
Male sex	336 (69.4%)	216 (67.1%)	120 (74.1%)	0.115
ICU admission categories				
Medical	371 (76.7%)	239 (74.2%)	132 (81.5%)	0.075
Scheduled surgery	39 (8.1%)	27 (8.4%)	12 (7.4%)	0.709
Emergency surgery	74 (15.3%)	56 (17.4%)	18 (11.1%)	0.070
Reasons for ICU admission				
Respiratory disease	259 (53.5%)	163 (50.6%)	96 (59.3%)	0.072
Gastrointestinal disease	59 (12.2%)	46 (14.3%)	13 (8.0%)	0.047
Neurological disease	49 (10.1%)	35 (10.9%)	14 (8.6%)	0.443
Cardiovascular disease	46 (9.5%)	27 (8.4%)	19 (11.7%)	0.237
Trauma	34 (7.0%)	28 (8.7%)	6 (3.7%)	0.043
Renal disease	26 (5.4%)	14 (4.3%)	12 (7.4%)	0.159
Miscellaneous	11 (2.3%)	9 (2.8%)	2 (3.7%)	0.277
Comorbidities				
No comorbidity	158(32.6%)	119(37.0%)	39(24.1%)	0.004
Hypertension	166(34.3%)	102(31.7%)	64(39.5%)	0.087
Diabetes mellitus	85(17.6%)	53(16.5%)	32(19.8%)	0.369
Coronary artery disease	83(17.1%)	53(16.5%)	30(18.5%)	0.571
COPD	80(16.5%)	48(14.9%)	32(19.8%)	0.176
Cancer	55(11.4%)	27(8.4%)	28(17.3%)	0.004
Hematologic malignancy	10(2.1%)	7(2.2%)	3(1.9%)	1.000
Organ transplantation	9(1.9%)	4(1.2%)	5(3.1%)	0.169
Chronic respiratory failure	73(15.1%)	44(13.7%)	29(17.9%)	0.219
Chronic heart failure	56(11.6%)	23(7.1%)	33(20.4%)	<0.001
Immunocompromise	48(9.9%)	25(7.8%)	23(14.2%)	0.025
Chronic renal failure	13(2.7%)	6(1.9%)	7(4.3%)	0.138
Chronic liver dysfunction	11(2.3%)	7(2.2%)	4(2.5%)	1.000
APACHE II, median (IQR)	21(16–27)	18(14–24)	25(19–32)	<0.001
SOFA on ICU day1, median (IQR)	7.5(5–10)	7(5–9)	9(6.75–12)	<0.001
Complications				
Septic shock	119(24.6%)	68(21.1%)	51(31.5%)	0.012
Acute kidney injury	201(41.5%)	119(37.0%)	82(50.6%)	0.004
ARDS	265(54.8%)	150(46.6%)	115(71.0%)	<0.001
ICU stay, days, median (IQR)	7(4–15)	7(4–14)	9(4–17)	0.067

APACHE II, Acute Physiology and Chronic Health Evaluation II; ARDS, acute respiratory distress syndrome; COPD, Chronic obstructive pulmonary disease; ICU, Intensive care unit; IQR, interquartile range; SOFA, Sequential Organ Failure Assessment.

### Sources of infection and microbiology

The lung (85.7%) and the abdomen (18.0%) were the most common sites of infection (Table 3). One hundred and sixty-seven patients (34.5%) had two or more infection sites. Only 37 patients (7.6%) had bloodstream infection.

**Table 3:** The source of infection of patients with severe sepsis (total >100% because 167 patients had more than one infection sites).

Variables	All patients (n = 484)	Survivors (n = 322)	Non-survivors (n = 162)	*P* Value
Pneumonia	419 (86.6%)	276 (85.7%)	143 (88.3%)	0.436
Intra-abdominal infection	80 (16.5%)	58 (18.0%)	22 (13.6%)	0.215
Gastroenteritis	41 (8.5%)	27 (8.4%)	14 (8.6%)	0.924
Urinary tract infection	37 (7.6%)	25 (7.8%)	12 (7.4%)	0.889
Bloodstream infection	37 (7.6%)	16 (5%)	21 (13%)	0.002
Soft tissue infection	34 (7.0%)	19 (5.9%)	15 (9.3%)	0.172
Central nervous system infection	23 (4.8%)	19 (5.9%)	4 (2.5%)	0.094
Multiple-site infection (≥2)	167 (34.5%)	111 (34.5%)	56 (34.6%)	0.983

Only half of the ICU (11/22) reported the microbiology, and 148 patients (30.6%) had microbiological documentations associated with severe sepsis and septic shock. Out of these 148 patients with microbiological results, Gram-negative bacilli were isolated in 111 patients (75.0%), and Gram-positive organisms were isolated in 32 patients (21.6%). Only six patients were diagnosed as invasive fungal infection or fungemia (4.1%). Forty-nine patients (33.1%) had polymicrobial (≥2 infection agents) infections. The most prevalent species were *Acinetobacter baumannii*, *Pseudomonas aeruginosa*, *Escherichia coli*, *Klebsiella pneumoniae*, methicillin-resistant *Staphylococcus aureus*, and *Stenotrophomonas maltophilia* (Table 4).

**Table 4:** Distribution of microorganisms isolated from 148 patients.

Microorganism	Total (n = 269)	ICU-acquired (n = 221)	Non-ICU-acquired (n = 48)
Gram positives	39 (14.5%)	34 (15.4%)	5 (10.4%)
Methicillin-resistant *Staphylococcus aureus*	17 (6.3%)	14 (6.3%)	3 (6.3%)
Coagulase-negative *Staphylococcus*	10 (3.7%)	8 (3.6%)	2 (4.2%)
* Enterococcus faecium*	8 (3.0%)	8 (3.6%)	0
* Enterococcus faecalis*	3 (1.1%)	3 (1.4%)	0
* Streptococcus viridans*	1 (0.4%)	1 (0.5%)	0
Gram negatives	168 (62.5%)	137 (62.0%)	31 (64.6%)
* Acinetobacterbaumannii*	38 (14.1%)	30 (13.6%)	8 (16.7%)
* Pseudomonas aeruginosa*	33 (12.3%)	27 (12.2%)	6 (12.5%)
* Escherichia coli*	26 (9.7%)	24 (10.9%)	2 (4.2%)
* Klebsiellapneumoniae*	25 (9.3%)	21 (9.5%)	4 (8.3%)
* Stenotrophomonasmaltophilia*	16 (5.9%)	12 (5.4%)	4 (8.3%)
* Proteus mirabilis*	5 (1.9%)	3 (1.4%)	2 (4.2%)
* Serratiamarcescens*	4 (1.5%)	3 (1.4%)	1 (2.1%)
Other Gram negatives*	21 (7.8%)	17 (7.7%)	4 (8.3%)
Fungi**	6 (2.2%)	6 (2.7%)	0
* Candida albicans*	1 (0.4%)	1 (0.5%)	0
* Aspergillus* spp.	4 (1.5%)	4 (1.8%)	0
* Pneumocystis*	1 (0.4%)	1 (0.5%)	0

*including Burkholderia cepacia, Chryseobacter iumindologenes, Enterobacter cloacae, Enterobacteraerogenes, and Serratialiquefaciens.

**fungal infection here refers to the invasive fungal infection and fungemia.

### Outcome of patients

Among the 484 patients included in the cohort, 139 died in ICU, and 23 died during hospitalization after transfer to general wards. Twenty patients (4.1%) were still in hospital at the end of follow-up (i.e. November 30, 2009) and were deemed survivors. The crude ICU and hospital mortality rates were 28.7% and 33.5%, respectively. The median ICU LOS was 7 days (IQR, 4–15) and hospital LOS was 18 days (IQR, 10–38). About three fourths (72.3%) of patients had stayed in ICU for less than 2 weeks, while 9.5% of patients had an ICU LOS of more than 4 weeks. Compared with patients without shock, patients with septic shock were more likely to receive mechanical ventilation (78.2% vs. 63.3%, p = 0.003), and had a longer ICU LOS (9 [IQR, 4–17.5] vs. 7 [IQR, 4–15], p = 0.011).

### Prognostic factors

Variables added into the multivariate model included age, sex, comorbidity of cancer, hypertension, COPD, and organ transplantation, chronic heart failure, immune-compromised status, type of ICU admission (respiratory disease, gastrointestinal disease, renal disease and trauma), ICU admission categories (medical, scheduled surgery and emergency surgery), APACHE II score, SOFA score of day1 in ICU, presence of septic shock, ARDS and AKI, bloodstream infection, soft tissue infection, central nervous system infection. As shown in Table 5, APACHE II score, presence of ARDS, bloodstream infection and comorbidity of cancer were independent risk factors for hospital mortality. The Chi-square value of Hosmer-Lemeshow test was 4.868, and the p value was 0.772.

**Table 5:** Multivariate logistic regression analysis of independent predictors of hospital mortality in patients with severe sepsis and septic shock.

Risk factor	OR (95% CI)		P value	
	Univariate	Multivariate	Univariate	Multivariate
APACHE II	1.113(1.083–1.144)	1.068(1.027–1.109)	<0.001	0.001
ARDS	3.173(2.129–4.730)	2.676(1.691–4.235)	<0.001	<0.001
Bloodstream infection	2.848(1.443–5.624)	2.520(1.142–5.564)	0.002	0.022
Cancer	2.283(1.295–4.024)	2.246(1.141–4.420)	0.004	0.019

APACHE II, Acute Physiology and Chronic Health Evaluation II; ARDS, Acute respiratory distress syndrome; CI, confidence interval; OR, odds ratio.

## Discussion

The incidence of severe sepsis in the present study was 37.3 cases per 100 ICU admissions, and 9.2% (n = 119) of the patients admitted into ICU developed septic shock. The lung and abdomen were the most frequent sites of infection. The ICU and hospital mortality rates of severe sepsis were 28.7% and 33.5%, respectively. Patients with shock had a much higher mortality rate (42.9%) than those without shock (30.4%). Patients with higher APACHE II score, presence of ARDS, bloodstream infection and comorbidity of cancer may have higher risk of death.

In comparison with previous studies [3], [6], [7], [21], the treated incidence of severe sepsis in the study ICUs is very high. This difference may be due to the increasing trend of severe sepsis [4], [5], as our study was conducted much later. However, secular trend may not be the only cause of incidence variation, because even higher incidence of severe sepsis has been reported in previous studies [9]. Different inclusion criteria may also explain the reported discrepancy. For example, Adrie et al included patients with ICU LOS of at least 48 hours [9], while Padkin et al only screened patients for severe sepsis within the first 24 hours of ICU admission [21]. In order to include cases with early recovery from or late onset of severe sepsis, we screened all ICU admissions with ICU LOS no less than 24 hours. Second, patient population in different studies may be quite different. Cheng et al only studied patients admitted to surgical ICUs [3], while, in the current study, 18 out of the 22 participating ICUs were general ICUs, and 76.7% of our cohort were medical patients. Another possible reason is the relative lack of ICU beds in China compared to other countries, which might have led to admission of sicker patients into the study ICUs [22], [23]. Finally, definitions of severe sepsis employed in various studies may be also different [21].

The outcome of severe sepsis patients varies considerably in different studies. In SOAP study, ICU mortality rate was 32.2% for severe sepsis and 54.1% for septic shock [24]. In France, patients with severe sepsis had a hospital mortality rate of 59% [8], whereas patients with septic shock had a hospital mortality of 61.2% [4]. Finfer et al reported that overall ICU and hospital mortality rates were 26.5% and 37.5% for patients with severe sepsis in Australia and New Zealand [6]. In comparison, the mortality rates of patients with severe sepsis (30.4%) and septic shock (42.9%) in the current study were lower than that in most studies [6], [12], [25]. Many factors can explain the difference. First, the severity of acute illness might be different. For example, patients included in the study of Khwannimit et al [12] were more severely ill, as suggested by a higher APACHE II score (26.8 vs. 21), and were more likely to die than our cohort (49.7% vs. 33.5%). Second, previous studies found that, compared with patients who developed sepsis outside ICU, patients with ICU-acquired sepsis had a higher mortality [24], [26]. Only 12.6% of patients in the current study developed ICU-acquired severe sepsis or septic shock, while 25% episodes of severe sepsis in France were ICU-acquired [8]. Despite the fact that hospital mortality rate in the current study was lower than that in many studies, it was still higher with a median APACHE II score of only 21. This was possibly attributable to the very low compliance with sepsis resuscitation and management bundles, although not reported in the current study but consistently observed in other studies involving Chinese patients [27]. There should be no doubt that clinical outcome of severe sepsis/septic shock could be improved significantly by better understanding of pathogenesis, as well as increasing the uniform compliance with standard therapy and other treatments proven effective for severe sepsis in the future. Due to the high mortality rate in patients with septic shock, patients with risk factors [28], [29], such as higher SOFA score should be observed closely.

Similar to other studies [4], [6], [8], we found that lung and abdomen were the most common source of infection. The potential implication of this finding is that, when the source of infection remains unknown in a patient with severe sepsis/septic shock, clinicians should consider pulmonary and intra-abdominal sources. Furthermore, majority of our patients had pneumonia, indicating the importance of implementing effective strategies to prevent both community-acquired pneumonia (such as public education, and vaccination against influenza and pneumococcus in high risk population) and hospital-acquired pneumonia (such as hand hygiene, and selective digestive decontamination). In accordance with other studies [3], [12], most isolated pathogens in our study were Gram-negative bacilli, although some studies in developed countries reported predominance of Gram-positive bacteria [2], [6], [24]. Many factors, including geographic variation, case mix, and antibiotic prescription habits, may explain the observed difference. Moreover, clinical significance of the same pathogens may vary in different studies. Cheng et al reported that *Acinetobacter baumannii* (25.8%) and *Escherichia coli* (13.8%) were the most common pathogens, while only 13.8% of the infections were caused by *Pseudomonas aeruginosa*. Despite a similar frequency of *Acinetobacter baumannii* (25.7%), *Pseudomonas aeruginosa* and *Klebsiella pneumonia* were much more common in our study. The dramatic increase in the incidence of *Pseudomonas aeruginosa* is worth mentioning as *Pseudomonas* species may be associated with increased mortality rates [24]. There might be bias in the results of microorganisms distribution as not all units reported microbiology results. Furthermore, we cannot rule out the possibility that many patients included in our study were nosocomial cases of sepsis, which might have resulted in the high frequency of *Acinetobacter baumannii*.

Independent risk factors associated with increased mortality in severe sepsis include higher APACHE II score [12], [27], presence of ARDS [12], [30], bloodstream infection [8] and comorbid cancer [2], [3], [24], [31], which was a consistent finding across literatures. Although patients with shock had a higher mortality, the presence of shock was not an independent risk factor for death in our study.

As we know, there are few data about the epidemiology of severe sepsis and septic shock in mixed ICU in China. Our report may add some valuable information. Nonetheless, some limitations merit discussion. First, there may have been bias only concerning ICU admissions. In general, patients with severe sepsis would be treated in ICU in those participating hospitals, unless they responded to simple measures such as fluid resuscitation and antibiotics. Some patients may not be admitted into ICU because of personal willingness. However, most Chinese people tend to reject advance directives, and prefer family-centered decision making than other ethnic and cultural groups. Even if the illness is irreversible, families may strongly advocate aggressive treatment, as they endorse the cultural belief that withdrawing or withholding support of their family member with critical illness is disgraceful or not filial piety [32]. Chinese doctors seldom advise families to withdraw treatment because of the possibility of involvement in medical disputes and undertaking legal liability [33]. Second, the results of the present study might not be able to generalize to ICUs in small local hospitals. Although about 10% (47/484) of our cohort are transferred from other hospitals, we believe that even more patients with severe sepsis/septic shock are treated in local hospitals, possibly with higher mortality rate. Third, only a minority of patients had microbiological documentation, and the rest were with negative or unreported positive culture results. But the distribution of pathogens obtained in this research was similar to the result of Cheng and colleagues' research [3], and there was no significant difference in patient demographics and mortality rate (38% vs. 31.3%, p = 0.144) between those ICUs with and without microbiological results. Zahar et al have also found that microbiological characteristics of infection did not influence the outcome of patients with severe sepsis [34]. Fourth, the exclusion criterion of ICU LOS<24 hours would have the effect of excluding the sickest patients who died early and might have bias the study results. Finally, the data were not collected for the primary purpose of identifying severe sepsis. Some important information related to severe sepsis was not reported, such as the compliance with sepsis bundle which might affect the clinical outcome of severe sepsis and the results of multivariate regression analysis. Further studies focusing on the rate of compliance with the resuscitation bundle and its influence factors should be conducted in China.

## Conclusions

Severe sepsis is an important public health problem and a frequent cause of ICU admission with a high mortality rate. Higher APACHE II score, presence of ARDS, bloodstream infection and comorbidity of cancer are risk factors contributing to fatal outcome. This argues that severe sepsis/septic shock represents a major disease burden in mainland China. Future clinical research is warranted to ensure early identification of high risk patient population, prompt implementation of validated treatment, and significant improvement of clinical outcome.

## Supporting Information

Appendix S1 **The full names and affiliation of participating hospitals.**
(DOC)
