# Supplementary material for: Epidemiology and Outcome of Severe Sepsis and Septic Shock in Intensive Care Units in Mainland China
Source: PLoS One. 2014 Sep 16;9(9):e107181. doi: 10.1371/journal.pone.0107181 (PMC4167333; doi:10.1371/journal.pone.0107181)
Supplement: Appendix S1 — The full names and affiliation of participating hospitals. (DOC) [file pone.0107181.s001.doc]

**The full names and affiliation of participating hospitals**

1. Medical ICU, Peking Union Medical College Hospital, Peking Union Medical College & Chinese Academy of Medical Sciences, Beijing, China
2. Department of Emergency Medicine and Medical ICU, The First Affiliated Hospital of Kunming Medical University, Kunming, China
3. Department of Critical Care Medicine, The First Affiliated Hospital of Harbin Medical University, Harbin, China
4. Department of Critical Care Medicine, First Affiliated Hospital, Xinjiang Medical University, Urumqi, China
5. Department of Critical Care Medicine, West China Hospital, Sichuan University, Chengdu, China
6. Department of Critical Care Medicine, The First Affiliated Hospital of China Medical University, Shenyang, China
7. Department of Critical Care Medicine, Xiangya Hospital, Central South University, Changsha, China
8. Department of Critical Care Medicine, Beijing Tongren Hospital, Capital Medical University, Beijing, China
9. Department of Critical Care Medicine, The Second Hospital of Jilin University, Changchun, China
10. Department of Critical Care Medicine, Peking University People’s Hospital, Beijing, China
11. Department of Critical Care Medicine, Qilu Hospital of Shandong University, Jinan, China
12. Department of Critical Care Medicine, Zhejiang Provincial People’s Hospital, Hangzhou, China
13. Department of Critical Care Medicine, Tongji Hospital of Tongji Medical College, Huazhong University of Science & Technology, Wuhan, China
14. Department of Critical Care Medicine, Hebei Medical University Fourth Hospital, Shijiazhuang, China
15. Department of Critical Care Medicine, General Hospital of Ningxia Medical University, Yinchuan, China
16. Department of Emergency and Intensive Care Medicine, The First Affiliated Hospital of Chongqing Medical University, Chongqing, China
17. Department of Critical Care Medicine, Fuxing Hospital, Capital Medical University, Beijing, China
18. Department of Critical Care Medicine, The First Affiliated Hospital of Fujian Medical University, Fuzhou, China
19. Emergency ICU, Ruijin Hospital, Shanghai Jiao Tong University, Shanghai, China
20. Department of Critical Care Medicine, Guangdong General Hospital, Guangzhou, China
21. Department of Critical Care Medicine, Hainan Provincial People’s Hospital, Haikou, China
22. Department of Critical Care Medicine, The Affiliated Hospital of Inner Mongolia Medical University, Huhhot, China
